# The Adult Mouse Anatomical Dictionary: a tool for annotating and integrating data

**DOI:** 10.1186/gb-2005-6-3-r29

**Published:** 2005-02-15

**Authors:** Terry F Hayamizu, Mary Mangan, John P Corradi, James A Kadin, Martin Ringwald

**Affiliations:** 1 The Jackson Laboratory, 600 Main Street, Bar Harbor, ME 04609, USA; 2 Current address: OpenHelix, 65 Main Street, Somerville, MA 02145, USA; 3 Bristol-Myers Squibb Pharmaceutical Research Institute, 5 Research Parkway, Wallingford, CT 06492, USA

## Abstract

We have developed an ontology to provide standardized nomenclature for anatomical terms in the postnatal mouse. The Adult Mouse Anatomical Dictionary is structured as a directed acyclic graph, and is organized hierarchically both spatially and functionally. The ontology will be used to annotate and integrate different types of data pertinent to anatomy, such as gene expression patterns and phenotype information, which will contribute to an integrated description of biological phenomena in the mouse.

## Abstract 2

The Adult Mouse Anatomical Dictionary was developed to provide an ontology for standardized nomenclature for anatomical terms in the postnatal mouse. The ontology will be used to annotate and integrate different types of data pertinent to anatomy.

## Rationale

An important role for biological databases is the integration of different types of data. Ontologies aim to overcome the semantic differences encountered in data collection and representation, providing common terminology in order to facilitate this integration. An anatomy ontology is a structured vocabulary of anatomical entities in which the terms have unique identities and relate to each other in meaningful ways. For many biological applications, anatomy ontologies are essential for standardized description of data directly related to anatomy, such as gene expression patterns and phenotype information.

The Gene Expression Database (GXD) is a resource for gene expression information from the mouse [1]. GXD has been designed as an open-ended system able to store and integrate primary data from many types of expression assays, each of which describe gene expression at different levels of spatial resolution. Currently, both GXD and the Edinburgh Mouse Atlas Gene Expression (EMAGE) database [2] use terms from the Mouse Embryo Anatomy Nomenclature Database [3] developed by the Edinburgh Mouse Atlas Project (EMAP) to describe patterns of gene expression in the developing mouse. However, since GXD also collects gene expression data from mice at postnatal stages, including adult, it became apparent that extension of GXD to fully annotate expression data for adult structures would require the development of a controlled vocabulary beyond the scope of the embryonic mouse anatomy ontology. Therefore, we developed an anatomy ontology for the postnatal mouse.

Critical to this effort was the realization that existing sources of controlled vocabularies for anatomy were not sufficient for use with the adult mouse, for several reasons. First, none conforms well to the structure of the embryonic mouse anatomy ontology created by our Edinburgh collaborators, an important factor in enabling planned integration between these ontologies (see below). Human-oriented anatomical ontologies have been developed (for example, the Foundational Model of Anatomy (FMA) [4], OpenGalen [5] and SNOMED CT [6], which covers human and veterinary medicine). In general, the complexity of the concepts represented by these ontologies, issues concerning their accessibility, as well as questions of relevance to the mouse, made it clear that they were neither well suited to nor adequate for our objectives. Thus, one of our goals was to follow the basic framework of the developmental ontology, while taking full advantage of the range of other resources available.

Another major consideration involved determination of the hierarchical structure and format of the ontology. Our experience using the developmental ontology made it clear that a mechanism to provide alternative hierarchies would be a critical factor. Consequently, the Adult Mouse Anatomical Dictionary is structured as a directed acyclic graph (DAG) in which an anatomical term can be represented as a child of more than one hierarchical parent term using both *is-a *and *part-of *relationships. The ontology is organized hierarchically in both spatial and functional ways, and contains more than 2,400 unique anatomical terms for the postnatal mouse. As GXD is part of the larger Mouse Genome Informatics (MGI) system, the ontology will also be used to annotate other types of data pertinent to adult mouse anatomy in order to provide an integrated description of a wide array of biological phenomena in the mouse.

## Developing an ontology for adult mouse anatomy

### Anatomical terms

GXD has extensive experience with the Mouse Embryo Anatomy Nomenclature Database, available through Theiler Stage (TS) 26, which is used by GXD and EMAGE to describe developmental gene expression patterns. Based on our annotation work, we continue to contribute to this ontology in the form of extensions and revisions, and by adding synonyms. Consequently, an early objective was to ensure that the anatomy ontology for the postnatal (TS 28) mouse corresponds as much as possible, both in content and in structure, with the developmental ontology. This was done for consistency of nomenclature, because we were familiar with and confident of the utility of this format, and to facilitate the future integration of these ontologies. Eventually, the goal is to combine and integrate the ontologies to generate an anatomy ontology covering the entire lifespan of the laboratory mouse.

With the developmental ontology as its framework, the effort was then focused on compiling an extensive list of anatomical terms for the postnatal mouse. The list was based on a number of major sources, including mouse atlases as well as anatomy and histology text resources [7-22]. For the most part, the preference was to focus on those that were mouse-specific. However, others that were more general were nevertheless extremely valuable. The non-atlas format references were especially useful in the effort to refine anatomic and histological details.

Once the basic list of terms had been generated, we confirmed that each term on the initial list represented actual mouse structures. These determinations were usually clear but at times ambiguous. For example, for numerous structures described in anatomy and histology textbooks, no clear documented evidence was found for their existence in the mouse. Consequently, these have not been included in the ontology. Further work is ongoing to ensure accuracy. Careful attention was paid to validating each term, with the requirement for two or more reliable sources whenever possible. Concurrent with the textbook-based identification of terms was the continuing effort to expand the vocabulary using a research data-driven approach. This method included extensive evaluation of published biomedical research literature, as well as data with anatomical attributes that have been collected in scientific databases. For example, several mouse-specific datasets [23-26] were used as resources to find pertinent anatomical terms. The MGI list of all mouse tissues from which major publicly available cDNA libraries have been generated [24] includes cell types and tumors, as well as gross anatomical concepts. The relevant anatomical structures will eventually be translated using terms from the Adult Mouse Anatomical Dictionary. The data-driven approach was especially useful in determining the level of granularity (that is, level of detail of spatial resolution) expected to be required by users of the ontology.

An additional consideration in determining the content of the vocabulary had to do with whether to include cell types. While cell type information is an important component in anatomical descriptions, this also introduces a level of complexity that is difficult to address adequately. We felt that it would be unfeasible to extend the representation to the cellular level owing to the large number of required hierarchical levels and leaf nodes. Therefore, it was concluded that the adult mouse anatomy ontology would not contain cell types, but that cell type terms would eventually be provided by the orthogonal controlled vocabulary for cell types currently being developed as part of the Open Biological Ontologies (OBO) effort [27]. However, to conform to the Edinburgh developmental ontology, we have included tissue type terms such as *epithelium *and *mesenchyme*, as well as defined cell type structures such as *purkinje cell layer*. In addition, we have also elected to include the term *unfertilized egg *and its synonyms.

### Hierarchical organization

The anatomy ontology for mouse development is currently structured as a straight hierarchy. In this format, an anatomical term can have only one parent and, thus, one place in the hierarchy. For example, the term *femur *is placed in the hierarchy according to this limb bone's spatial location, as a substructure of the *upper leg*, rather than as a part of the *skeleton*. In contrast, the *brain *is described as being part of the *central nervous system*, rather than as a part of the *head*. Based on our experience with the developmental ontology and anticipating planned revisions for it, we decided to represent the adult mouse anatomy ontology as a DAG, in which a given anatomical term is able to have more than one hierarchical parent. This allowed us flexibility in organizing the hierarchies, and provided a mechanism to create a more comprehensive view of the relationships between the anatomical terms.

**Figure 1:**
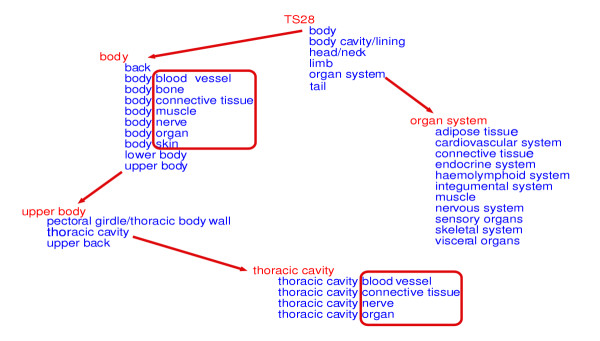
Hierarchical organization of the adult mouse anatomy ontology. The hierarchy is divided into spatial and organ system components. Blocks indicate generic group terms appropriate to multiple spatial regions.

For each of the anatomical terms being evaluated, any one of a number of pathways to that term could be conceptualized. However, it also soon became apparent that two fundamental characteristics could be determined for most of the terms: its spatial location within the animal and its functional contribution as part of a particular organ system. Consequently, we decided to use the distinction between spatial versus organ system representation as an organizational principle. Since 'spatial part' does not itself represent a unique anatomical entity, it was not included as an independent node in the ontology. However, the initial division of the hierarchy into spatial and organ system components is immediately apparent in the first level of substructures below the root node, TS28. As shown in Figure 1, this level is predominantly comprised of spatial parts: for example *body*, *body cavity/lining*, *head/neck*, *limb *and *tail*. Accordingly, terms defined by these superstructures are primarily organized according to spatial localization. In contrast, another branch of the hierarchy is indicated by the superstructure *organ system*, where the anatomical terms are organized, as much as possible, according to their respective contribution to a specified functional system.

Currently, the distinction between spatial and functional relationships is represented only implicitly. However, based on the parentage of anatomical structures, biologists will be able to intuitively discern both types of relationships. Furthermore, they should be able to perform most of the queries related to expression and phenotype data that are currently envisioned. Explicit representation of both relationship types might be a desirable feature for advanced knowledge representation and computational analysis. On the other hand, it might also introduce unnecessary complexities to a biologist because, for example, many anatomical structures would have both spatial and functional relationships between them. Shielding the user from those complexities would require additional software development. A careful evaluation of the advantages and disadvantages of both approaches will direct our future work in this area.

During the construction of the adult mouse anatomy DAG, we had to take into account the fact that terms representing some tissues would logically be spatially located in numerous parts of the ontology. Groups of tissues which meet this criteria include: *blood vessel*, *bone*, *connective tissue*, *muscle*, *nerve*, *organ *and *skin*, which are represented as terms in the *organ system *part of the hierarchy. To accommodate the need to represent these tissues in specific body regions, we devised modules (outlined as blocks in Figure 1) representing these generic groups. These have been included as subterms, when appropriate, within each spatial region. For nomenclature standardization (more on this below), the subgroup terms are preceded by superstructure name, in noun form (that is, *abdomen*) rather than as an adjective (for example, abdominal) whenever possible.

**Figure 2:**
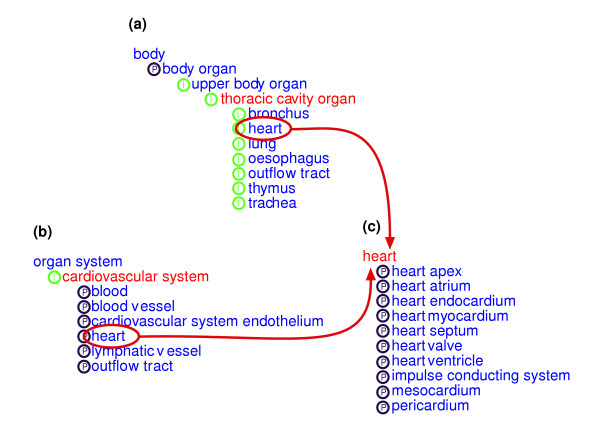
Example showing multiple hierarchical representations for a given anatomical term. The *heart *is represented both as **(a) **an organ in the thoracic cavity, and **(b) **as a part of the cardiovascular organ system. **(c) **Detail page for the term *heart *showing immediate substructures. Note that both spatial and functional representations contain *is-a *and *part-of *relationships.

Consequently, using the DAG format, we have been able to describe adult mouse anatomy from a variety of spatial and organ system perspectives. For example, the *heart *(Figure 2) is represented as a type of *thoracic cavity organ*, as well as a substructure of the *cardiovascular system*. As will be discussed below, some of these distinctions are conceptual and by their nature may be somewhat arbitrary. However, from our annotation work we know that the different breakdowns of the anatomy are indeed required to annotate, for example, different types of expression and phenotype data. It should be emphasized that refinements to the hierarchical organization of the ontology will continue to be made. These changes will not affect the identity of the terms themselves.

Another issue in constructing the DAG was the use of *is-a *and *part-of *relationships between the terms. Overall, most of the relationships could be classified intuitively as *part-of*, indicating that the term is a component of the more general term above it in the tree. For example, the upper *body *is considered to be *part-of *the *body*, and the heart is *part-of *the *cardiovascular system*. In contrast, *is-a *relationships are used to indicate that an anatomical term represents an instance of the certain type or kind of the concept denoted by its parent term. For instance, the *cardiovascular system is-a *specific *organ system*, while *cardiac muscle is-a *type of *muscle*. It should be noted that there is no correlation between the *is-a *and *part-of *relationships and the spatial versus *organ system *organization of the ontology, as shown in Figure 2. Further refinement of the relationships will undoubtedly be required, as well as additional types of relationships. For example, it may be useful to distinguish between 'regional' parts (for instance, head, neck, limb) versus 'systemic' parts (for instance, body muscle, body organ, body skin). These modifications can be easily accomplished using the DAG-Edit tool (see Software section below).

### Nomenclature considerations

Our experience with the mouse developmental ontology, as well as extensive literature review, provided the primary basis for the naming conventions that were employed. Early in building the ontology, we realized that consistent nomenclature, not only for a given term itself but for related terms and groups of terms, would be a critical requirement. Consequently, whenever possible, the same name was used for a given anatomical structure or concept throughout the ontology. For instance, we have used the term *lung *rather than 'pulmonary' to precede each of the terms representing lung substructures. Another consideration regarded the need to clearly distinguish between terms. It is theoretically possible to precisely define an anatomical term based on a combination of the term name and the hierarchical lineage of the term. The term *epithelium*, for example, is represented as a subterm for many anatomical structures, and a given term's precise identity could be defined by its parental lineage. From a practical standpoint, this convention has proved to be problematic; multiple structures with the same term name would be impossible to distinguish in absence of its hierarchical context. This would be complicated further by any additional pathway to a given term. For instance, *epithelium *of the lung alveoli is represented both as a part of the *alveolus *and as a type of *lung epithelium*. To address this issue, we have attempted to provide sufficient information in the term name (for example *alveolus epithelium*) so that it becomes easy to interpret and use the term unambiguously.

Other factors that were considered were the requirements of the DAG-Edit software (see below), as well as features promoting unambiguous identification of terms. Additional conventions employed for the naming of anatomical terms included: structure names are preceded by superstructure names, in noun form; terms are used in singular form, whenever possible; all term names at the same level in the hierarchy are ordered alpha-numerically; and all characters are in lower case. Nomenclature consistency will also facilitate querying for specific anatomical terms within the ontology.

### Software issues

An ontology should contain a level of detail appropriate to the data being classified and the level at which queries are likely to be performed, while simultaneously providing sufficient flexibility to enable regular updating without needing to significantly modify the hierarchies. Therefore, we recognized that the adult mouse anatomy ontology would require a format that was both robust and flexible, as well as the tools to accommodate the need for maintenance and updating. The DAG-Edit tool developed by the Gene Ontology (GO) Consortium provides a graphical interface to handle any vocabulary that has a DAG data structure, and has been used by other groups to build ontologies for a wide range of biological subjects, including the GO [28] and Mammalian Phenotype ontology [29]. We have utilized DAG-Edit both for construction of the adult mouse anatomy ontology and for maintenance and editing. Furthermore, the MGI software group has developed a range of tools to handle a DAG-formatted ontology, enabling navigation through the ontology and querying for terms (see below), as well as integration of the ontology with other information stored within the MGI database.

## Current status and future directions for the Adult Mouse Anatomical Dictionary

**Figure 3:**
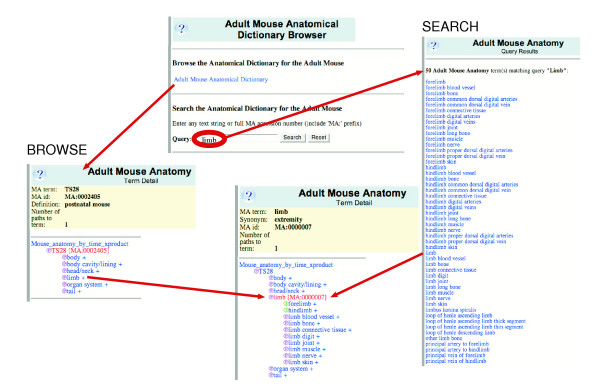
Using the Adult Mouse Anatomical Dictionary Browser. The MGI Browser allows the user to either browse (progressively navigate through the various hierarchies) or search (enter a text string to query for terms, for example *limb*) within the adult mouse anatomy ontology. 'Term Detail' pages include the unique numerical identifiers (that is MA ID numbers) for each term, as well as relevant definitions and/or synonyms.

We have developed an ontology containing more than 2,400 unique terms to provide standardized nomenclature for anatomical structures in the postnatal mouse. The Adult Mouse Anatomical Dictionary can be accessed at the MGI web site [30]. The MGI Browser page (Figure 3) enables one to navigate through the ontology in two ways. Browsing results in the display of progressively lower levels in the hierarchy. Information about individual terms, including its relationship to other terms in the hierarchy, is shown in a 'Term Detail' page. Alternatively, one can search the ontology by using the 'Query' field, which accepts any text string and searches for all terms in the vocabulary, including any synonyms, containing that string. The resulting 'Query Results' page displays all structures that match the query, and also provides links to the appropriate 'Term Detail' page. The adult mouse anatomy ontology can also be viewed and downloaded at the OBO website [31]. The ontology can be saved in several different formats including GO flat file and OBO formats, as well as XML/RDF and OWL.

We will continue to expand and refine the Adult Mouse Anatomical Dictionary in response to additional sources of information, as well as the needs of the scientific community. As part of the ontology's ongoing development, we plan to: expand the list of terms, based on additional resources as they become available; further edit the hierarchies when necessary; and provide alternative names for terms as synonyms. A limited number of synonyms have already been included (for example, see 'Term Detail' page for *limb *in Figure 3). It is envisioned that many more will be added as required, which will also aid in querying for specific terms in the ontology. Precise definitions for each of the terms will also be included as appropriate. Eventually, the adult mouse anatomy ontology will be merged with the Anatomical Dictionary for Mouse Development to generate an anatomy ontology covering the entire lifespan of the laboratory mouse. The proposed effort will include representation of *derived-from *types of relationships linking anatomical structures at subsequent developmental stages. Such relationships will allow querying for progenitor and derivative tissues. These associations will also enable analysis of differentiation pathways, thus enhancing the ability to explore biological phenomena occurring in the mouse.

Anatomy vocabularies are being developed for other organisms and there has been interest in integrating these ontologies at some level. One such effort is the XSPAN project [32], which aims to support cross-species interoperability between developmental anatomy ontologies. On a different scale, Standards and Ontologies for Functional Genomics (SOFG) [33] has set up an international effort to integrate anatomy ontologies of mouse and human. A recent project has been development of the SOFG Anatomy Entry List (SAEL) [34], a list of commonly used anatomical terms that will be directly linked to several major anatomy ontologies, particularly those for human and mouse. It is envisioned that this list will serve as a controlled vocabulary to describe low-resolution anatomical attributes of biological data. For example, the terms included have sufficient resolution to distinguish most samples used for microarray experiments. The Microarray Gene Expression Data (MGED) ontology will use the SAEL for describing anatomical attributes of mouse microarray data. The SAEL and the MGED ontology will also serve as entry points to more comprehensive anatomical resources such as the Adult Mouse Anatomical Dictionary.

The Adult Mouse Anatomical Dictionary will be used as a resource to enable standardization and integration of many types of biological data pertinent to postnatal mouse anatomy, including expression, biological process, phenotype and pathology data. GXD currently uses terms from the ontology to annotate expression information at all postnatal stages. While expression results are currently annotated using an abridged version, efforts are underway to map expression data directly to the expanded adult mouse anatomy ontology. GO project curators use terms from the anatomy ontology to describe mouse anatomical concepts. The Mouse Genome Database (MGD) incorporates or associates relevant terms from the adult anatomy ontology into the Mammalian Phenotype Ontology, which is being developed to provide standard terms for annotating mouse phenotype data. Eventually, the standardized anatomy terms will be used to directly link gene expression and phenotype annotations within MGI via the anatomy. The mouse anatomy ontology is also being used to annotate phenotype data for the Eumorphia project [35]. In Pathbase, a database of mutant mouse pathology [36], anatomical attributes of images for mutant postnatal mouse pathology are coded using terms based on the Adult Mouse Anatomical Dictionary. Furthermore, efforts are currently underway to incorporate the adult mouse anatomy ontology into the National Cancer Institute (NCI) Thesaurus, a knowledgebase containing the working vocabularies used in NCI data systems [37].

Anatomy is an important biological integrator. Like expression data, many biological processes and phenotypic observations relate to specific anatomical structures. We have successfully promoted the idea that such data should be described using the same anatomical descriptors. Specifically, we have shown that this can be achieved by describing more complex types of biological information in a modular fashion by combining terms from orthogonal vocabularies [38]. The combinatorial approach takes advantage of existing terms and relationships in the base ontologies. This approach is now being used by most of the resources and projects mentioned above. The use of common anatomical terms will allow for a direct integration of expression, biological process and phenotypic data in the mouse. Links provided with the anatomy terms will, for example, allow display of both expression data and phenotype information associated with specific anatomical structures in the anatomical dictionary browser, as is already the case for developmental expression data [23]. Furthermore, this type of integration will enable complex queries that directly correlate expression and phenotype data. For example, the system will allow queries such as "Which mouse mutants display phenotypes in a specific anatomical structure?" and "How does gene expression in this anatomical structure, or in precursors of this anatomical structure, differ between these mutants and wild type animals?" Answers to these types of queries hold the promise of providing direct insights into the molecular mechanisms underlying differentiation and disease.
